# Pathological images for personal medicine in Hepatocellular carcinoma: Cross-talk of gene sequencing and pathological images

**DOI:** 10.32604/or.2022.027958

**Published:** 2023-02-03

**Authors:** LI YANG, KUN DENG, ZHIQIANG MOU, PINGFU XIONG, JIAN WEN, JING LI

**Affiliations:** 1 Department of General Surgery (Hepatobiliary Surgery), The Affiliated Hospital of Southwest Medical University, Luzhou, 646000, China; 2 Academician (Expert) Workstation of Sichuan Province, Luzhou, 646000, China; 3 Department of General Surgery (Gastrointestinal Surgery), The Affiliated Hospital of Southwest Medical University, Luzhou, 646000, China

**Keywords:** Hepatocellular carcinoma, tumor environment, individualized medicine, predictive model, pathological model

## Abstract

**Background:**

Considering the great heterogeneity of Hepatocellular carcinoma (HCC), more accurate prognostic models are urgently needed. This paper combined the advantages of genomics and pathomics to construct a prognostic model.

**Methods:**

First, we collected data from hepatocellular carcinoma patients with complete mRNA expression profiles and clinical annotations from the TCGA database. Then, based on immune-related genes, we used random forest plots to screen prognosis-related genes and build prognostic models. Bioinformatics was used to identify biological pathways, evaluate the tumor microenvironment, and perform drug susceptibility testing. Finally, we divided the patients into different subgroups according to the gene model algorithm. Pathological models were constructed by obtaining HE-stained sections from TCGA in corresponding subgroups of patients.

**Results:**

In this study, we constructed a stable prognostic model that could predict overall survival in HCC patients. The signature consisted of six immune-related genes (*BX537318.1, TMEM147, CSPG4P12, AC015908.3, CEBPZOS*, and *SRD5A3*). We found increased levels of infiltration of immune cells in the tumor microenvironment in patients with low risk scores, indicating significant antitumor immunity and corresponding to better clinical outcomes. We then screened nine drugs that were more sensitive in the low-risk group than in the high-risk group. Finally, we addressed the complex cellular changes and phenotypic heterogeneity in the HCC microenvironment by combining genomics and pathomics analysis methods.

**Conclusion:**

Our study showed that the prognostic evaluation model of HCC based on the immune signaling pathway is feasible and provided a reference value for potential immunotherapy for HCC.

## Introduction

Globally, the incidence and mortality of primary liver cancer are the sixth and fourth highest, respectively, among which hepatocellular carcinoma (HCC) accounts for 75%–85% of primary liver cancer [1–3]. Liver is the sixth most common site of primary cancer in humans, often in the context of cirrhosis and inflammation. Cholangiocarcinoma is the second most common primary liver malignancy after HCC [4–6]. In recent decades, the incidence of cholangiocarcinoma has increased significantly, with a 5-year survival rate of <10% [7]. Although patients with early liver cancer can be surgically resected or given a liver graft, most patients with primary liver cancer are too advanced to be eligible for surgical resection. In addition, owing to its anatomical location and tissue, as well as its unique metabolic and immunosuppressive environment, the liver is often colonized by cancer metastases from other organs [8,9]. In recent years, tumor immunotherapy has achieved good results in many cancers, but the therapeutic effect of immunotherapy in liver cancer has not been satisfactory [10,11]. Under physiological conditions, tumors accelerate cancer development by avoiding immune responses [12–14]. The key regulatory node of immunotherapy is the immuno-tumor microenvironment, which is divided into the T-cell infiltration-exclusion type, infiltration-inflammatory type and lymphoid structure infiltration type [13–15]. A better understanding of the immune microenvironment is an important prerequisite for immunity against liver cancer [16].

Tumors resist immune responses by activating immune checkpoints, such as programmed death-1 (PD-1) and its ligand PD-L [17–19], as well as cytotoxic T-lymphocyte-associated protein 4 (CTLA-4) [20,21]. Immune checkpoint inhibition uses monoclonal antibodies targeting PD-1/PD-L1 and CTLA-4 to release pre-existing immunity, particularly effector CD8^+^ T cells [13,22,23]. Immunotherapy has a positive effect on some patients but has no obvious effect on most patients [24,25]. Owing to the adaptability of tumors to different microenvironments, different individuals have different abilities to respond to treatment. Therefore, accurate immunotherapy and predictive biomarkers are important to achieve the best therapeutic effect and prolong the quality of life of patients. Exploring new immune-related prognostic markers is important for guiding the treatment process and prolonging the survival of patients with liver cancer.

Cancer diagnosis, prognosis, and prediction of response to therapy are often done using heterogeneous data sources, including histological sections, molecular profiles, as well as clinical data, such as patient age and comorbidities [26]. Histology-based subjective and qualitative analysis of the tumor microenvironment combined with quantitative examination with genomic testing is the standard of care for most cancers in modern clinical settings [27–29]. As the field of anatomic pathology moves from slides to digitized whole-slide images, it becomes possible to analyze pathomics and genomics in a comprehensive manner. Current modern sequencing technologies such as single-cell sequencing are capable of dissecting genomic information from individual cells in tumor specimens, and spatial transcriptomics and multiplex immunofluorescence are capable of spatially dissecting histological tissue and genomics together [30–33]. However, these techniques currently lack clinical penetration The Cancer Genome Atlas (TCGA) contains genomically-paired whole-slide images, genotype and transcriptome data from cancer patients with ground truth survival and histological grading markers, providing a powerful tool for the combination of pathomics and genomics [34].

In our investigation, we analyzed liver cancer data from The Cancer Genome Atlas (TCGA) database, selected immune-related prognostic biomarkers, and then constructed predictive model. However, we have not stopped yet. Because pathological imaging plays an important role in revealing the tumor microenvironment, it is limited by the complexity of previous multi-genomics studies and has not been fully applied to the analysis of liver cancer. With the rapid development of biomedical imaging applications in cancer, pathology combined with multi-genomics showed great application potential in liver cancer prediction. This is the first original study to combine pathological images and genetic groupings to complement each other.

## Methods

**FIGURE 1:**
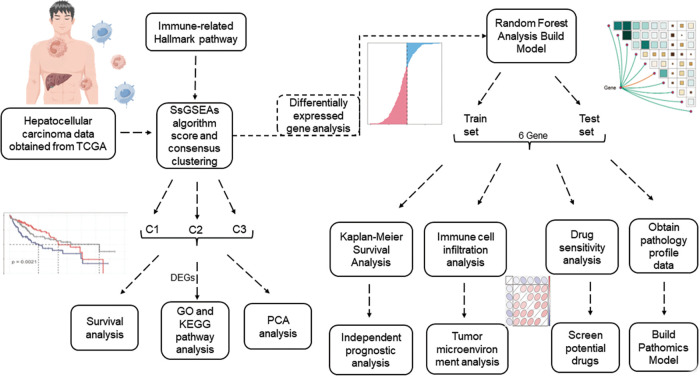
Flowchart.

Flowchart was found in Fig. 1.

### Data acquisition

The TCGA (Cancer Genome Atlas) database was created by the National Cancer Institute and contains genomic, transcriptomic, proteomic, and methylation data from 20,000 primary cancers (http://cancergenome.nih.gov/). Transcriptomic data and corresponding clinical information were collected from a total of 368 HCC patients by screening samples that were not eligible for data or had missing clinical data.

### Single-sample gene set enrichment analysis (ssGSEA)

Immune-related hallmark pathways were included in this study. The ssGSEA algorithm was used to score, and consensus cluster analysis was performed to identify immune-related patterns [35]. The R package ConsensusClusterPlus (Version 1.56.0) was used with key parameters, including maxK = 6 and repetition = 500 for stable identification [36]. A total of 368 patients with HCC were divided into three groups by consensus clustering. We further performed differential expression gene (DEG) analysis using the R package LIMMA (version 3.48.3) to compare pairwise expression of different patterns of genes using the LMFIT and EBayes functions to ensure accuracy. DEGs were selected according to the standard; the adjusted *p*-value was less than 0.001, and the absolute value of logFC was more than 1.

### Immune-related signaling pathways

Gene Ontology (GO) annotation analysis was performed using the R package clusterProfiler (version 4.0.5) with a false discovery rate (FDR) of less than 0.05 to determine significant enrichment [37]. GSEA Version 4.1.0 (Broad Institute, Cambridge, MA, USA) was used to identify a predefined genomic cohort *p*-value of less than 0.05, and an FDR of less than 0.25 for alterations between clusters 1‒3 in a consensus cluster were considered statistically significant [38].

### Construction of genome models

Based on the TCGA‒LIHC cohort, we used random forest analysis to screen for genes with prognostic markers from the target gene pool and constructed a prognostic model. Next, we distinguished the high-risk group from the low-risk group according to the median risk score.

### Assessment of immune cell infiltration

Two methods were used to calculate the immune infiltration score: ssGSEA and the xCell algorithm, which were visualized by stacking map correlation heat map and scatter plot, respectively.

### Extraction of pathological features

The Cancer Genome Atlas (TCGA) contains genomics paired whole-slide images, genotype, and transcriptome data from cancer patients with ground truth survival and histological grading markers [34]. To observe the histological changes and pathological changes in patients at high and low risk of LIHC, we obtained hematoxylin and eosin (H&E) stained sections from TCGA from each LIHC patient, and the patients to whom each section belonged corresponded to the genomics data of LIHC patients. We used CellProfiler software (3.0.0) downloaded from the CellProfiler website (www.cellprofiler.org), pathological images were analyzed, and pathomics feature extraction and data processing were performed using a special pipeline set by ourselves [39,40]. A workflow for LIHC tumor pathology feature extraction was established by adding a pre-programmed algorithm module to the pipeline. The pipeline is available from public data (https://cellprofiler.org/published-pipelines).The specific pipeline is set as follows: We construct eight Gaussian filters, and the extracted feature types include texture feature, wavelet feature and pipeline feature. After a series of superposition operations, the constructed pipeline can extract 6564 pathological and histological features in each pathological image. In pathological images of LIHC patients, we obtained HE staining images of five typical patients from previously identified high-risk and low-risk groups of LIHC patients for feature extraction for histopathology. For each patient with LIHC, the most typical 10 PATCH images (256 × 256 size) were selected and each pathological image was magnified 20-fold in WSI format to obtain the corresponding PATCH images. Initially, biopsy margins, tears, or gaps within the tissue were excluded and applied to masked RAW images. Masked RAW images were then converted to grayscale to identify stained objects (DAB and nuclei). A threshold algorithm was applied to identify DAB and HTX (kernel) stained objects, respectively. Eventually, we obtained 50 images from each of the low-risk and high-risk groups for feature extraction and subsequent analysis.

### Construction of pathological model

For 100 pathological images obtained from LIHC patients, we randomly selected 70 training datasets (39/31 = positive/negative) as pathomics models. The other 30 served as independent test datasets (11/19 = positive/negative). To eliminate imbalances in the training dataset, we used a variety of data normalization and regularization methods and analyzed the degree of dominance of the respective constructed models in terms of prediction level. A normalization method was applied to the feature matrix. For each feature vector, we calculated the number of l2 parameters and divided them by. The feature vector is then mapped onto the unit vector. For the normalization method, we chose the minmax, z-score, and mean methods. Because of the high dimensionality of the feature space, principal component analysis (PCA) and Pearson correlation coefficient dimensionality reduction were applied to the feature matrix. The feature vectors of the transformed feature matrices are independent of each other. Before building the model, we used ANOVA, KW, RFE, and mitigation methods to select pathomorphologic features. These methods are often used to explore salient features corresponding to labels. Ultimately, we used logistic regression to construct a pathological risk model for the selected optimal pathological features. The prediction performance of the constructed pathological feature model was analyzed and evaluated using receiver operating characteristic curves in the training, validation, cross-training, and cross-validation groups. In addition, we assessed the goodness-of-fit and clinical applicability of the pathologic signature model using the Hosmer–Lemnshow test.

### Statistical analysis

The R software was used for all analyses. Student’s *t*-test was used for normally distributed continuous variables. Mann–Whitney U test was used for continuous variables that were not normally distributed. Limma package was used to analyze the differentially expressed threshold setting of logFC > 0.5, *p* < 0.05.

## Results

### Identification of immune pathway-related genes in HCC

**Figure 2:**
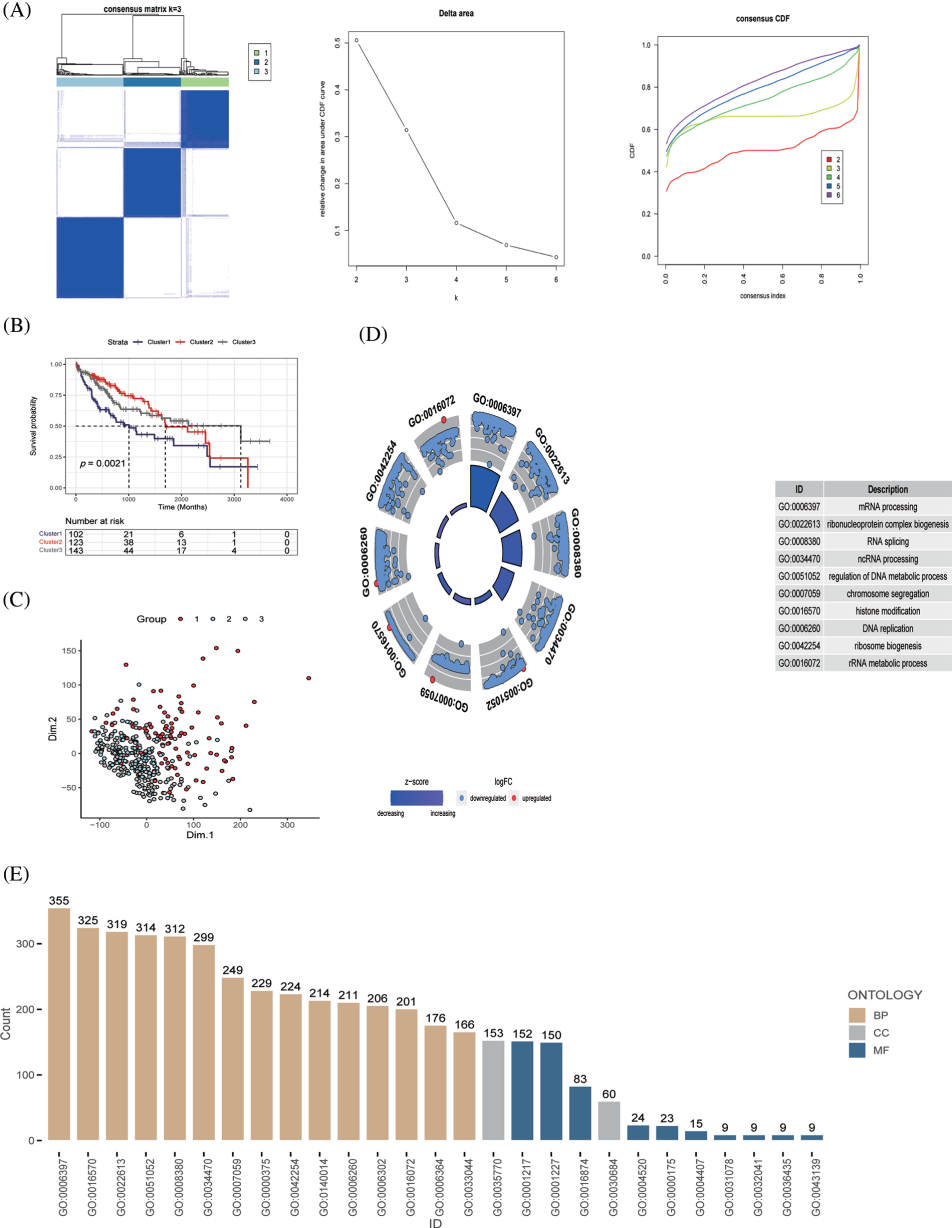
Identification of immune pathway-related genes and molecular subtype (A) Patients with HCC (*n* = 368) were classified into three groups by consensus clustering (K = 3); categorical evaluation of the three subsets formed after cluster analysis, (B) survival analysis of patients with differential subsets, and (C) distribution evaluation of subsets by PCA. (D, E) GO analysis of immune pathway-associated genes in liver cancer, discovering the cellular distribution of each gene and the functional association of gene expression.

First, we used 368 patients with HCC to identify gene patterns related to immune pathways by ssGSEA algorithm scoring and consensus clustering analysis and set the K value of the consensus matrix to 3. The patients were then divided into three groups according to the optimal conditions, with significant differences between the groups (Fig. 2A). K–M analysis revealed significant differences in survival among the three groups; patients in cluster 1 had the worst survival outcomes (Fig. 2B). PCA also revealed remarkable differences in gene expression distribution among the three subsets, demonstrating the success of our classification approach (Fig. 2C). To further evaluate the differences among the three subsets, we evaluated the functional status and quality of life of these genes *in vivo* using GO analysis based on the DEGs (Figs. 2D and 2E). From these results, we observed that the screened genes were highly correlated with the GO:0006397 pathway. These results suggest that based on the cohort information of the patients with HCC and the expression levels of genes related to immune signaling pathways, the patients were effectively divided into different molecular subgroups by consensus clustering analysis, and the effectiveness of classification was confirmed by PCA analysis.

### Functional analysis of immune-related genes

**Figure 3:**
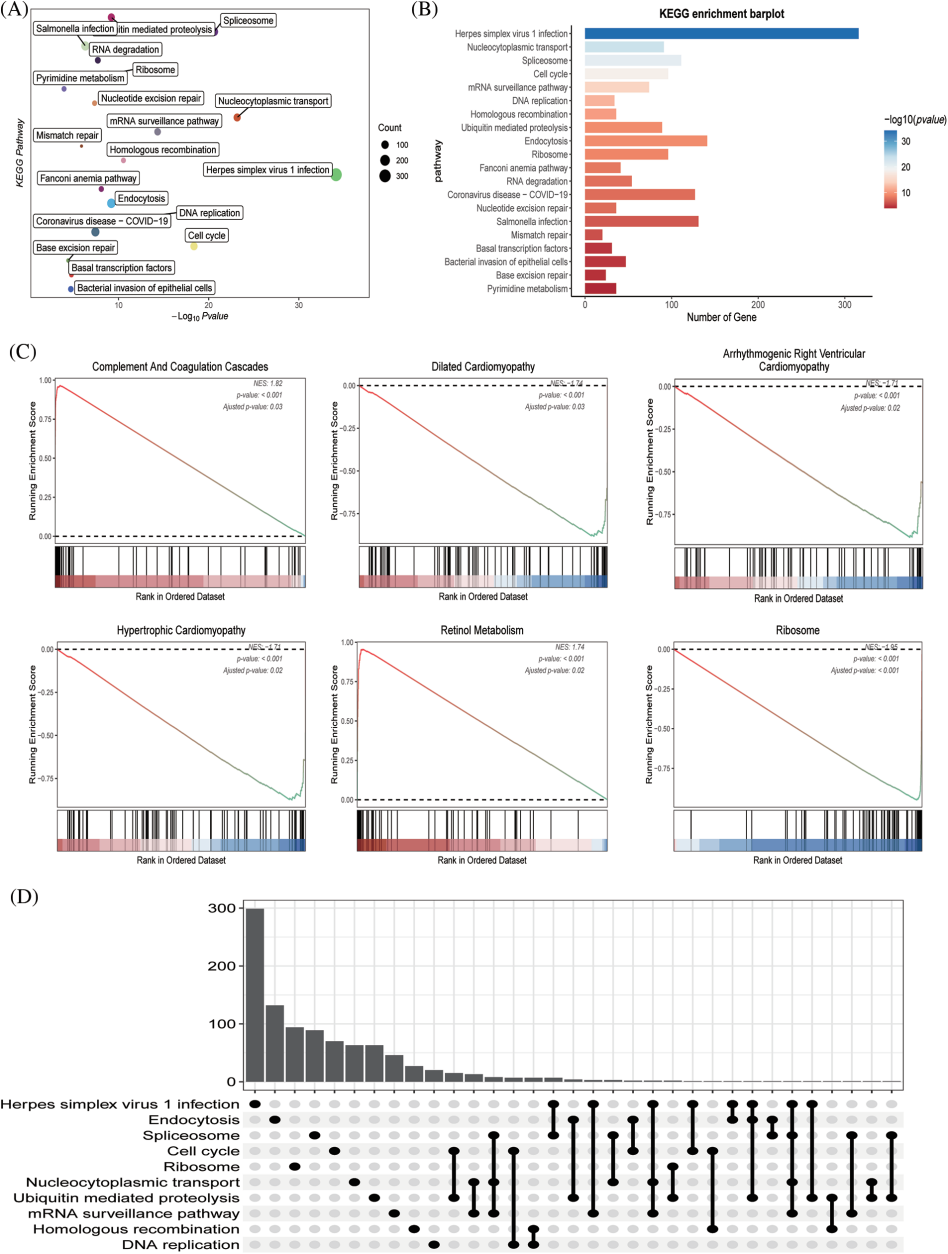
The functional assessment (A) KEGG pathway analysis of immune pathway-associated genes in liver cancer. (B) The number of genes involved in the enrichment pathway. (C) KEGG analysis revealed that metabolic and tissue developmental elements, including hypertrophic cardiomyopathy, ribosome, retinol metabolism, complement and coagulation cascades, dilated cardiomyopathy and arrhythmogenic right ventricular cardiomyopathy, were significantly associated with the immune pathway-associated genes. (D) The UpSet graph showing the genes involved in the pathway.

Through KEGG and functional enrichment analyses, we further confirmed the functional role and metabolically active pathways of the DEGs in tumor progression (143 genes). Fig. 3A displays that these DEGs were enriched in these pathways (cell cycle, DNA replication, herpes simplex virus 1 infection, and bacterial invasion of epithelial cells), the specific information was marked, and Fig. 3B showed the enriched gene number in the corresponding pathway. Subsequently, we further evaluated the relationship between these metabolic pathways and the genes of immune pathway, then found that hypertrophic cardiomyopathy, ribosome, retinol metabolism, complement and coagulation cascades, dilated cardiomyopathy, and the HCC immune model were significantly expressed (Fig. 3C). Fig. 3D represented an overview of the pathways involved in the genes.

### Identification of genes associated with HCC risk model

**Figure 4:**
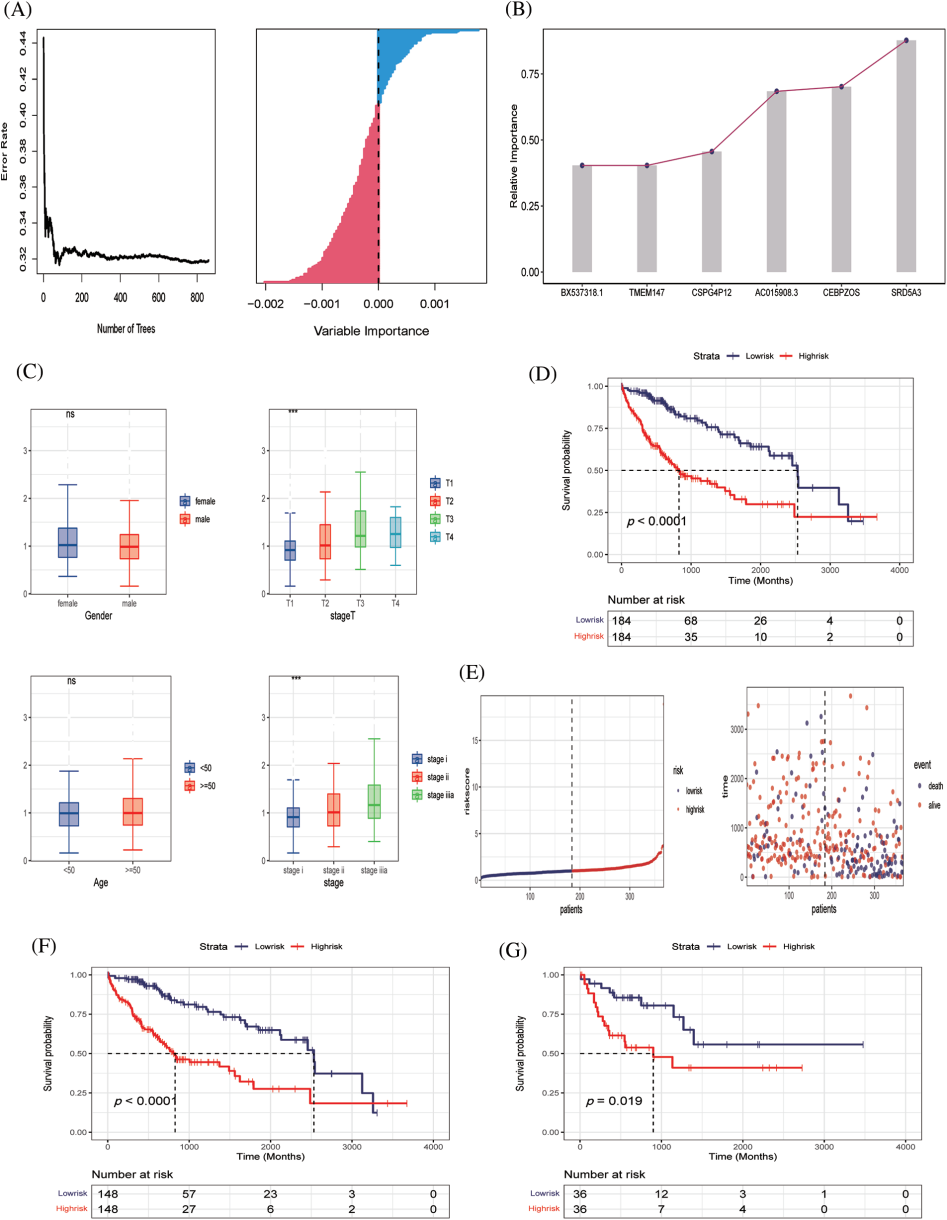
Construction of predictive model (A, B) Six genes of prognostic significance were selected from 143 genes based on LASSO regression to construct predictive models. (C) Risk values for patients with different clinical parameters, including gender, stage T, age, stage. (D) K‒M survival analysis curves of TCGA‒LIHC cohort. (E) Survival time and survival status distribution between high- and low-risk groups of the prediction model in the TCGA‒LIHC cohort. (F, G) K‒M survival analysis in training and validating cohorts.

Six genes of prognostic significance were selected from 143 genes in the TCGA–LIHC cohort according to a random forest algorithm: *BX537318.1, TMEM147, CSPG4P12, AC015908.3, CEBPZOS*, and *SRD5A3*, and prognostic models were constructed (Figs. 4A and 4B). Patients were divided into high-risk and low-risk groups according to the median risk score. We found that the risk score was independent of age and gender but correlated with T stage and clinical stage, and the risk value increased with disease progression (Fig. 4C). According to Kaplan–Meier (K–M) analysis, poor survival outcomes were evident for patients in the high-risk group (Fig. 4D). As the risk value increases, the patient ’s survival status becomes progressively worse (Fig. 4E).

### Preliminary evaluation of HCC risk model

To further verify the effectiveness of our prognostic model, we needed to draw survival curves based on the other cohort (internal cohort verification), which was divided 8:2 into training and validation sets. K‒M analysis and log-rank tests were used to compare the *p*-values of survival curves between the two groups (Figs. 4F and 4G). In both the training and independent validation sets, we found that patients with high-risk scores had poorer overall survival (*p* < 0.05), indicating the validity and accuracy of our prognostic model.

### Evaluation of tumor immune microenvironment based on the two algorisms

**Figure 5:**
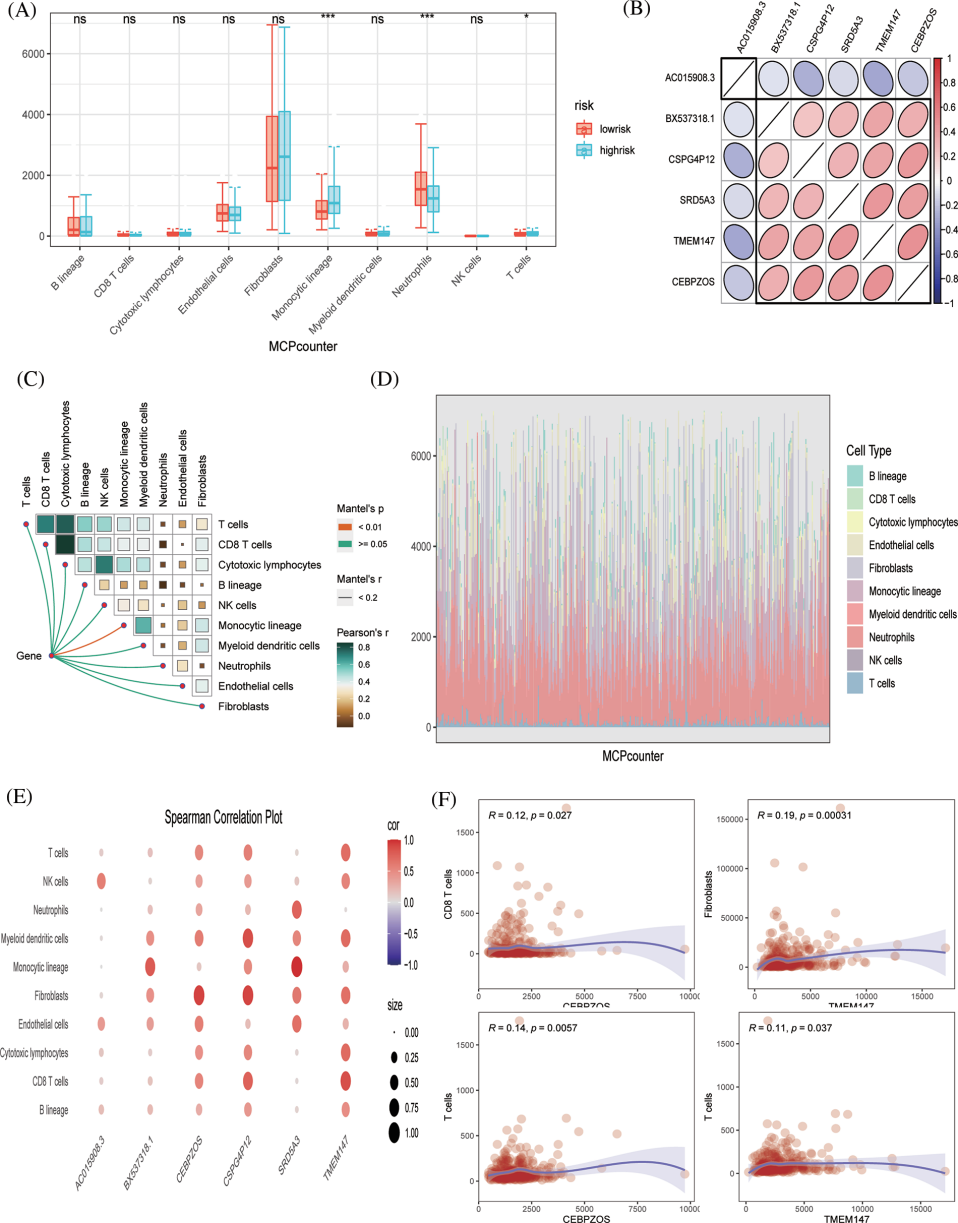
Immune infiltration evaluation (A) MCPcount algorithm constructed a box line plot of the difference in infiltration levels of several immune cells between the low-risk and high-risk groups in the predictive model. (B) Spearman correlation plots displaying the relationship among six prognostic genes. (C) Correlation of genes and immunocytes. (D) Stacked plots of the distribution of 10 major immune cells in the low-risk and high-risk groups. (E) Spearman correlation plots further visualized and clarified the relationship between the six genes and the specific cell types of immune infiltration. (F) The scatterplot showing the relationship between specific genes and immune cells.

Subsequently, because tumor immune infiltration plays a key role in tumor development and progression, we compared the differences in infiltrating immune cells between high-risk and low-risk groups by MCPcount algorithm analysis. The results showed that neutrophil infiltration was higher in the low-risk group, indicating that neutrophils may play an important role in anti-tumor immunity. Fig. 5B shows the correlation between the six prognostic genes. *AC015908.3* was negatively correlated with other genes, while other genes were positively correlated. Butterfly plots showed correlations between genes and immune cells (Fig. 5C). Fig. 5D shows the proportion of tumor cell infiltration in each patient. Bubble plots showed the association of six prognostic genes with immune cell infiltration (Fig. 5E). TMEM147 was positively associated with fibroblast production, *CEBPZOS* with CD8 + T cells, and *CEBPZOS* and *TMEM147* with T cells (Fig. 5F).

**Figure 6:**
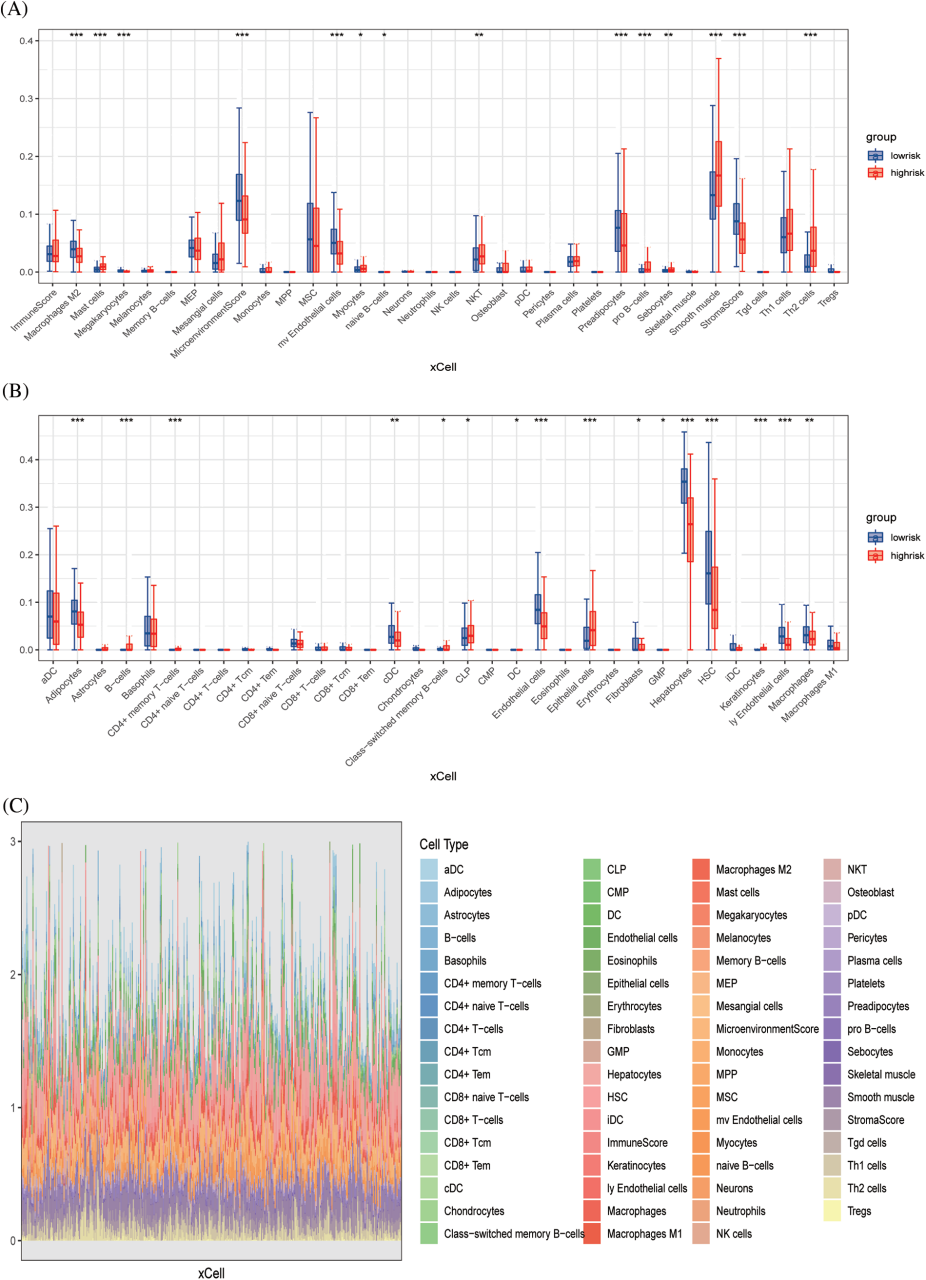
Immune infiltration validation (A, B) Boxplots showing differences in immune cell infiltration between high- and low-risk groups. (C) Stacked plots of the distribution of 50 major immune cells in the high- and low-risk groups.

**Figure 7:**
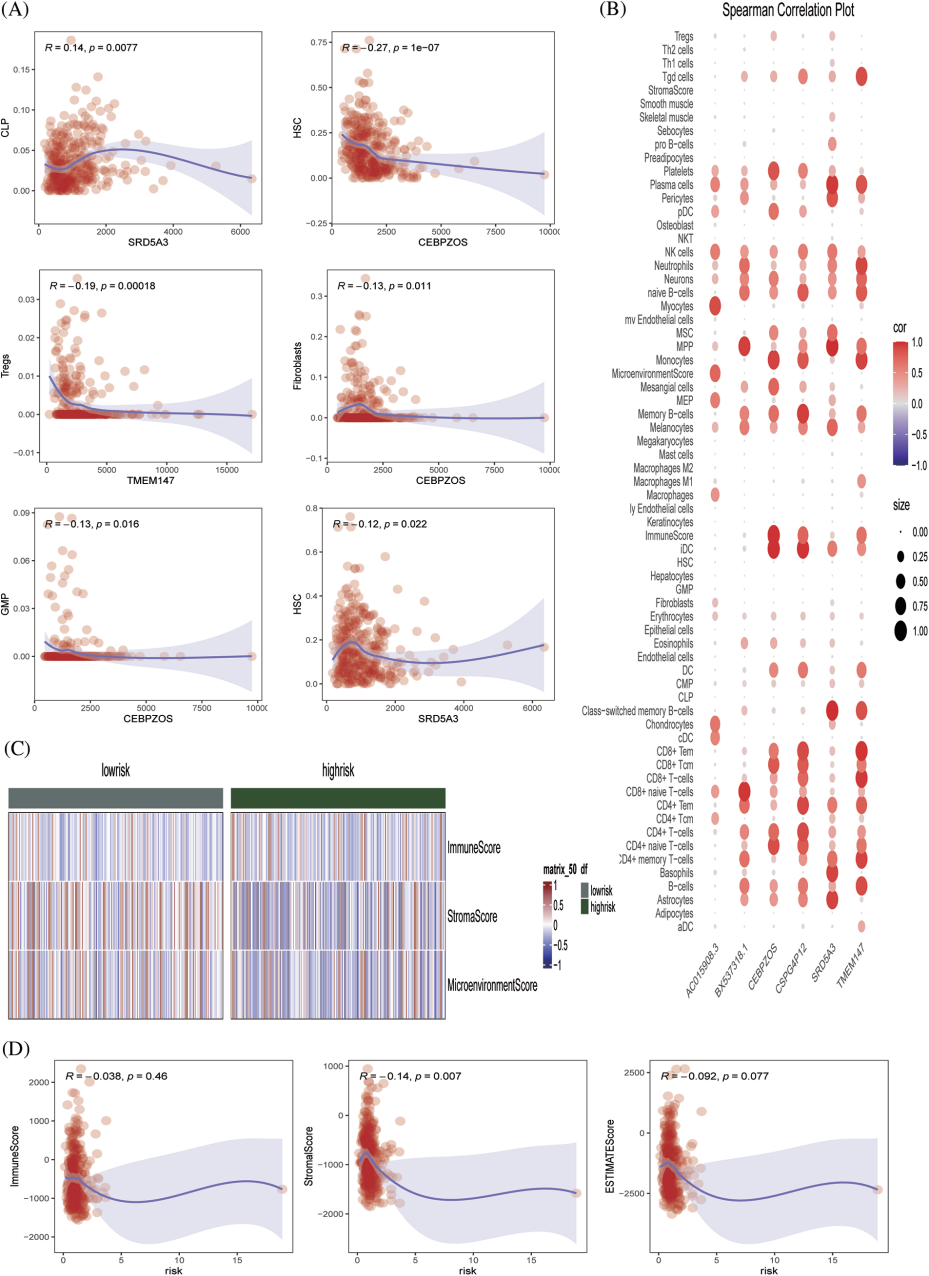
Immune infiltration validation (A) Scatterplot showing the relationship between specific genes and immune cells. (B) Spearman correlation plots further visualized and clarified the relationship between the six genes and the specific cell types of immune infiltration. (C) Heat map showing the immune scores of patients in the high- and low-risk groups. (D) Scatterplot showing the relationship between score and ImmuneScore, StromalScore, and ESTIMATE score.

The xCell algorithm was used to verify the above results. Similarly, patients in the low-risk group had an increased component of immune cell infiltration and an enhanced immuno-antitumor effect (Figs. 6A and 6B). Fig. 6C showed the percentage of immune cell infiltration in each tissue. *CEBPZOS* was positively correlated with GMP and fibroblasis, but negatively correlated with hematopoietic stem cell (HSC); *SRD5A3* was positively correlated with CLP and negatively correlated with HSC, and *TMEM137* was negatively correlated with regulatory T cells (Fig. 7A). The bubble graph shows the association of the six prognostic genes with immune cell infiltration (Fig. 7B).

The ESTIMATE algorithm was used to calculate the ESTIMATEScore, StromalScore, and ImmuneScore of each sample, and the heat map showed the high- and low-risk grade distribution subgroup patients (Fig. 7C). Fig. 7D showed that risk is negatively correlated with the ESTIMATEScore, StromalScore, and ImmuneScore. Conclusively, we preliminarily evaluated the effectiveness of the prognostic model by drawing survival curves and correlation verification, laying a solid foundation for further verification.

### Drug sensitivity analysis

**Figure 8:**
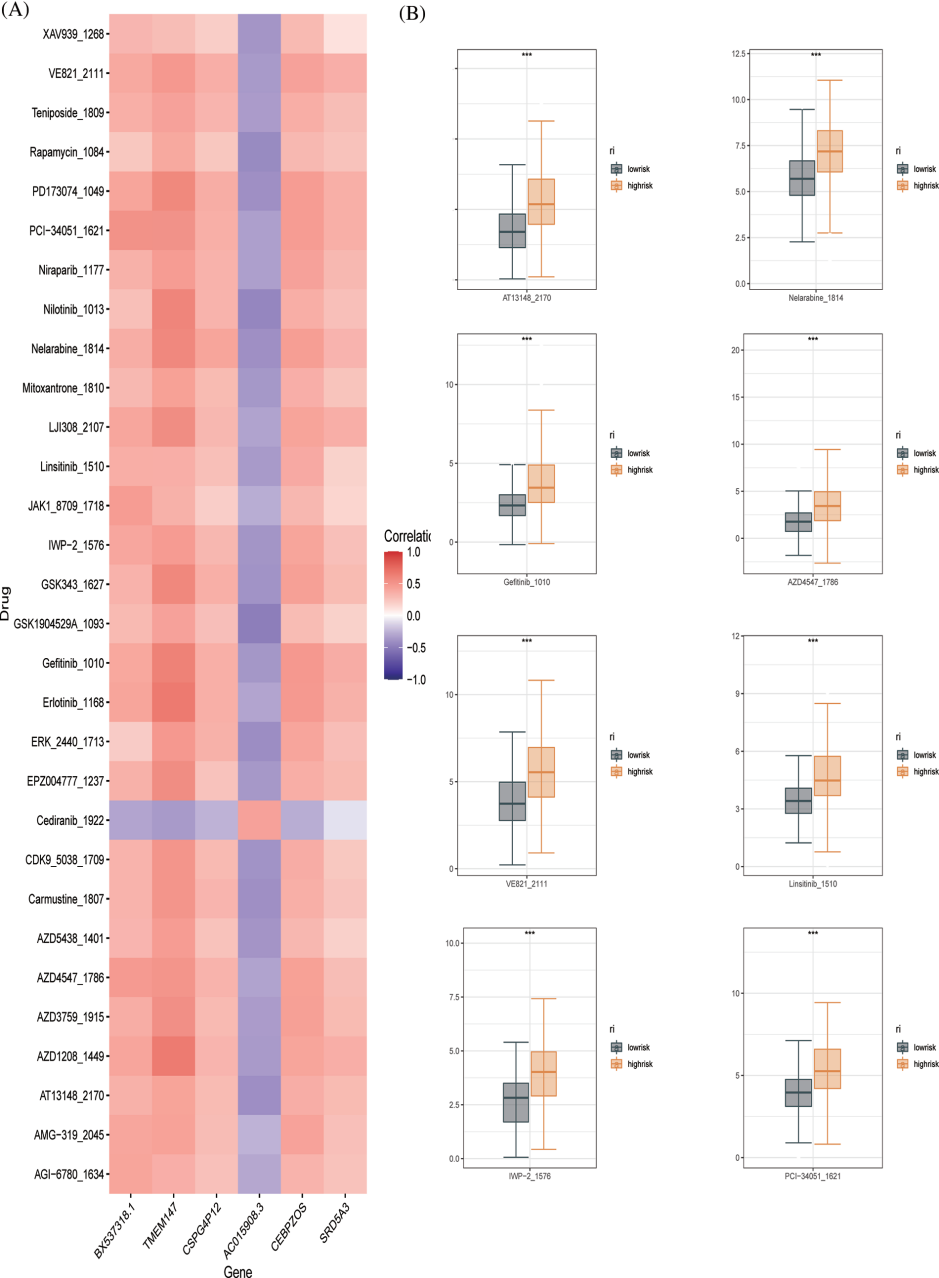
Drug sensitivity evaluation (A) Heat map displaying the correlation between six genes and therapeutic sensitivity of multiple chemotherapeutic drugs. (B) Box line plots showing significant therapeutic sensitivity between high- and low-risk subgroups for PD173074, PCI-34051, IWP-2, Linsitinib, VE821 AZD4547, Gefitinib, Nelarabine, and AT13148.

To further evaluate the drug sensitivity of the immune-signaling pathway-based prognostic assessment model for HCC, we introduced drug-calculated sensitivity scores from the GDSC database to screen potential therapeutic agents for HCC based on prognostic genes. We showed the susceptibility of different drugs to six differential genes in the form of a heatmap (Fig. 8A). Finally, we screened nine drugs that were more sensitive in the low-risk group than in the high-risk group, including PD173074, PCI-34051, IWP-2, linsitnib, VE821, PD173074, PCI-34051, IWP-2, VE821, AZD4547, gefitnib, nelarabine, and AT13148 (Fig. 8B). In conclusion, our prognostic evaluation model for HCC based on immune signaling pathways has good performance for drug prediction.

### Construction of pathological model

**Figure 9:**
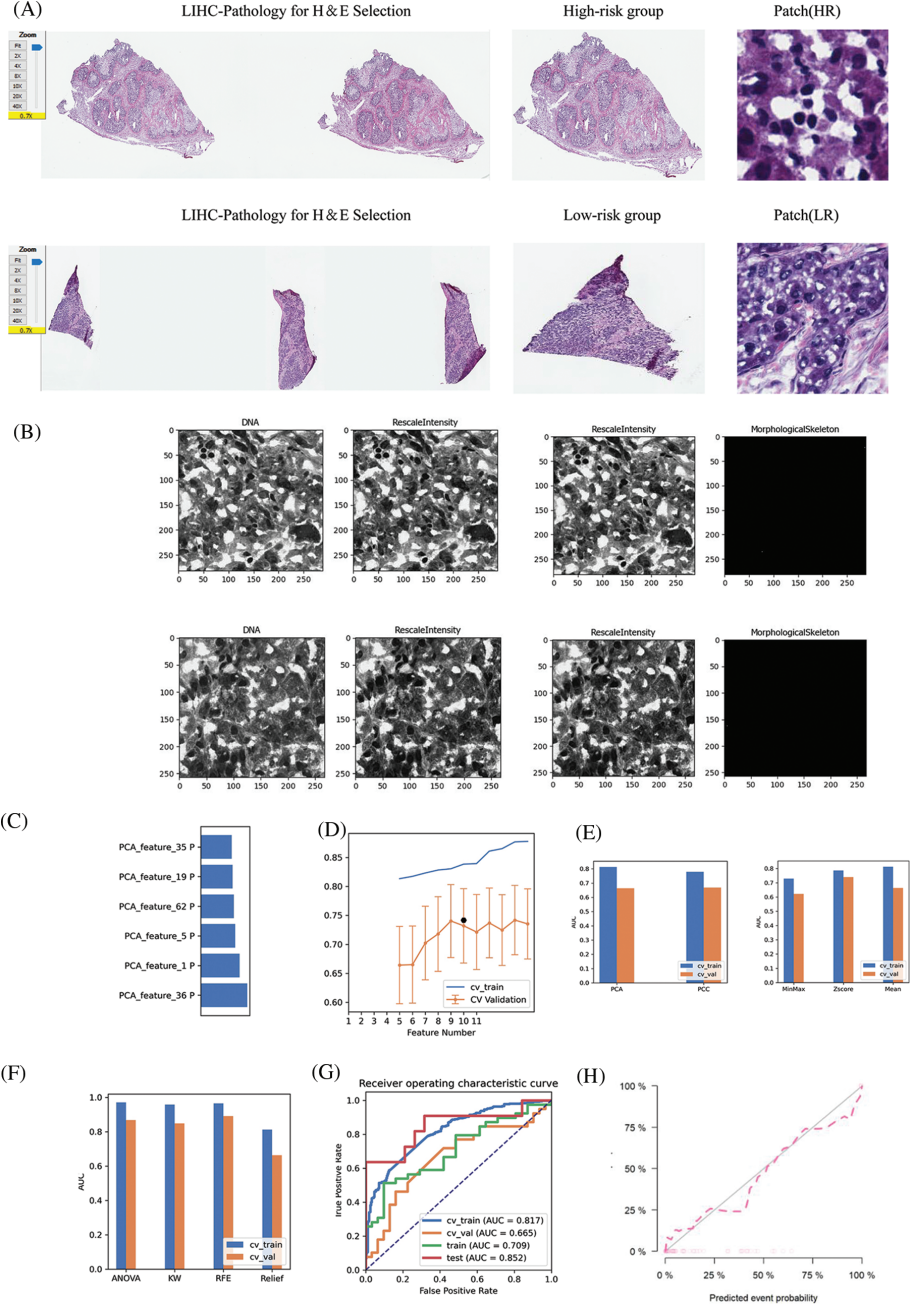
Pathomic marker mining and risk prediction model construction for LIHC. (A) HE-stained sections corresponding to patients with LIHC in the high- and low-risk groups screened in the pathology section repository; specific pathological images and patches outlined in the region of interest; (B) DNA, RescaleIntensity intensity analysis maps obtained from pathomics analysis. (C) Types of pathomic features constituting the pathomic risk model and their respective weights in the final model. (D) Linear analysis plot of the effect of the number of pathomic features on the AUC values of the final model. (E) Bar graph showing the effect of the selected dimensionality reduction method and data normalization method on the predictive efficacy of the pathomic risk model. (F) Analysis of the effect of different pathomic feature selection methods on the prediction of the pathomic risk model accuracy. (G) Histogram of the impact analysis of the constructed pathomics models on prediction ROC curves (training, validation, CV-training, and CV-validation groups, respectively). (H) Goodness-of-fit test curves for pathomics model.

The HE-stained sections corresponding to the high-risk and low-risk groups of patients with LIHC were screened against the pathology database; the specific pathological images and the region of interest patches are outlined in Fig. 9A. We obtained multiple pathomic features through a variety of pathology section feature extraction pipelines, including those within the DNA and skeletal regions, as shown in Fig. 9B. The weights occupied by the selected pathomic features in the machine learning model are shown in Fig. 9C. Differences in the area under the curve (AUC) values were similarly generated for different numbers of features in the same model (Fig. 9D). The differences in model prediction performance due to methodological differences between steps in the pipeline constructed through multiple normalization, feature selection, and dimensionality reduction methods to filter the optimal model are shown in Figs. 9E and 9F. The machine learning model obtained by the following method was selected to achieve the best AUC value and was therefore considered to be the best model for pathomics model construction and optimal pipeline formation. The feature vectors of the transformed feature matrix were independent of each other. Before building the model, we used Relief to select the features. Relief selects a sub-dataset and recursively finds the relative features according to the label. Logistic regression with the LASSO constraint was used as the classifier. Logistic regression with LASSO constraint is a linear classifier based on logistic, and the hyper-parameters were set according to the model performance on the validation dataset. The AUC and accuracy of the model were 0.852 and 0.867, respectively, for the test dataset (Fig. 9G). In the H-L test (Fig. 9H), we also demonstrated the excellent goodness-of-fit of the constructed LIHC pathomics model, demonstrating its clinical applicability.

## Discussion

Immunotherapy has a wide application prospect for cancer, but recent studies and clinical guidance have shown that its efficacy in liver cancer is much lower than that in other cancers. From the perspective of multi-genomics, we identified prognostic markers of HCC based on immune signaling pathways and explored the direct or indirect effect of prognostic markers of HCC and immunotherapy to provide more reference for the potential application of effective immunotherapy.

Significant progress has also been made in immunotherapy for liver cancer. Studies have reported that nivolumab against PD-1, a fully human immunoglobulin G4 monoclonal anti-PD-1 antibody, was first tested for safety and efficacy in a phase 1/2 study of more than 250 patients with advanced HCC [41]. The expanded cohort confirmed a good safety profile, with 19% of grades 3 and 4 treatment-related adverse events consisting primarily of transient laboratory abnormalities. Nivolumab was subsequently granted accelerated approval by the US Food and Drug Administration in September 2017 for the treatment of patients with advanced HCC previously treated with sorafenib [42,43]. Several other monotherapy immune checkpoint inhibitors targeting the PD-1/PD-L1 axis, including atezolizumab (NCT04157985), durvalumab (NCT03847428), toripalimab (NCT03949231), and tislelizumab (NCT03412773), also showed an immune response against liver cancer [10,44–48]. Cell-based immunotherapies, such as tumor-infiltrating lymphocytes or cytokine-induced killer cells (CIKs) for adoptive cell transfer, have potent antitumor effects with little cytotoxicity to normal cells. The efficacy and safety of activated CIKs in adjuvant therapy were evaluated in 230 patients with HCC, who underwent surgical resection, radiofrequency ablation (RFA), or percutaneous ethanol injection [49]. When there is a high endogenous tumor antigen load in the primary tumor, immunotherapy in the neoadjuvant setting can enhance T-cell priming and potentially eliminate micrometastases, which may be the source of postoperative tumor recurrence [47,50–52].

In this study, we first performed differential gene analysis based on immune signaling pathways in 368 patients with HCC from the TCGA‒LIHC cohort. By consensus cluster recognition, we divided patients into three clusters and further analyzed the expression of differential genes. GO and KEGG functional annotations were used to demonstrate that differential genes shared specific functional processes with immune signaling pathways. Six genes with prognostic significance, including *BX537318.1, TMEM147, CSPG4P12, AC015908.3, CEBPZOS* and *SRD5A3*, were selected from 143 genes according to random forest algorithm to construct a prognostic model, and the whole set was cut into 8:2---training set and validation set cohorts. The high- and low-risk groups were distinguished by the median risk score, and the two groups had significant prognostic differences. The ssGSEA and xCell algorithms were also used to calculate the immune infiltration score, which was visualized by a stacking correlation heat map and scatter plot, respectively.

Studies have shown that *TMEM147* interacts with the lamin B receptor in the endoplasmic reticulum, affecting the expression level and localization of the receptor [53], and *TMEM147* can also promote the proliferation of prostate cancer [54]. Studies have suggested that *CEBPZOS* is a prognostic marker of liver cancer related to energy metabolism [55]. Loss or overexpression of *SRD5A3* is closely related to breast cancer and loss of glycosylation function [56–58]. However, studies of these genes in liver cancer are relatively rare. We demonstrated that *SRD5A3* plays a critical role in HCC progression.

In recent years, more and more researchers have established cancer prognostic models based on novel cell death mechanisms, but they are missing in clinical applications [59]. Linking pathomics and genomics is a whole new direction, and this approach links the performance of models from theory to practice and is essential for performance validation before clinical application [27,60]. Recently, an increasing number of researchers have linked pathomics and genomics. In one study, researchers revealed the landscape of m6A methylation modification patterns in bladder cancer by radiogenomics mapping by linking pathomics and genomics [61]. Another study validated this approach using glioma and clear cell renal cell carcinoma datasets from The Cancer Genome Atlas (TCGA), which contains paired whole-slide images, genotype, and transcriptome data with survival and histological grading markers, providing strong help for pathomics and genomics linkages [27]. In our study, we combined the model with clinical findings by obtaining pathological patterns corresponding to patients in the high and low model groups by TCGA. For the future clinical treatment, we provide a reliable route, based on our model to diagnose the patient’s risk, through clinical biopsy to determine the patient’s disease progression, and finally based on the risk score, and finally choose the corresponding drug for treatment.

In summary, we constructed a stable prognostic marker that can predict the overall survival of patients with liver cancer by analyzing HCC-related data in the TCGA database. In addition, we used a combination of bioinformatics and pathomics analyses to elucidate the complex cellular and phenotypic heterogeneity in the HCC ecosystem; the information obtained from these studies is critical for the development of successful therapies. The complex timing of HCC with diverse immune cell subsets and extensive tumor-immune cell crosstalk also emphasizes the need for combination therapeutic approaches targeting these components. As a next step, we will combine experimental and clinical data to further clarify the clinical feasibility of this prognostic marker. Immunotherapy has great potential in the early stages of the disease, and several trials investigating the effectiveness of immune-based approaches in neoadjuvant and adjuvant settings are ongoing. Finally, the development of biomarkers that can effectively predict the response to immunotherapy is essential for identifying optimal therapeutic targets and selecting the appropriate treatment for each patient.

Our study still has some limitations. Our study is based on data from the TCGA platform linking genomic and pathological group outcomes in patients, which leads to challenging validation of our model. Although we have an internal validation set and cross-validation, it is better if there are external validations that can satisfy both genomic and pathological groups. In addition, our model also has its certain advantages. Compared with the previous conventional model, the model combined with clinicopathologic pattern has more application conditions, and is no longer a blank talk for predicting the prognosis of patients. It can find the differences between patients from the pathological point of view.

Conclusively, our study shows that the prognostic assessment model of HCC based on the immune signaling pathway is feasible and provides a reference value for potential immunotherapy for HCC treatment.

## Data Availability

All datasets generated for this study are included in the public database.
